# Supplemental Plant Extracts From *Flos lonicerae* in Combination With *Baikal skullcap* Attenuate Intestinal Disruption and Modulate Gut Microbiota in Laying Hens Challenged by *Salmonella pullorum*

**DOI:** 10.3389/fmicb.2019.01681

**Published:** 2019-07-24

**Authors:** Wei-wei Wang, Hong-jie Jia, Hai-jun Zhang, Jing Wang, Hui-yuan Lv, Shu-geng Wu, Guang-hai Qi

**Affiliations:** ^1^Risk Assessment Laboratory of Feed Derived Factors to Animal Product Quality Safety of Ministry of Agriculture and Rural Affairs, National Engineering Research Center of Biological Feed, Feed Research Institute, Chinese Academy of Agricultural Sciences, Beijing, China; ^2^Centre Technology Co., Ltd., Beijing, China

**Keywords:** gut microbiota, intestinal disruption, laying hen, plant extract, *Salmonella pullorum*

## Abstract

Dietary inclusions of baicalin and chlorogenic acid were beneficial for intestinal health in pigs. Nevertheless, it is unknown whether these plant-derived products had protection for intestine against bacterial challenge in chickens. This study was aimed at evaluating the potential mitigating effects of plant extracts (PE) from *Flos lonicerae* combined with *Baikal skullcap* (the active components are chlorogenic acid and baicalin) on intestinal disruption and dysbacteriosis induced by *Salmonella pullorum* in laying hens. A total of 216 41-week-old layers were randomly divided into 3 groups (6 replicates per group): negative control (NC), *S. pullorum*-infected positive control (PC), and the *S. pullorum*-infected group with supplementation of PE at 1000 mg/kg. All birds except those in NC were challenged with *S. pullorum* at the end of 4 weeks of the experiment. *S. pullorum* challenge impaired (*P* < 0.05) the production performance (egg production, feed intake, and feed efficiency) of laying hens, increased (*P* < 0.05) serum endotoxin content and frequency of *Salmonella*-positive organs, as well as up-regulated (*P* < 0.05) ileal expression of pro-inflammatory cytokines including *IFNG*, *TNFA*, *IL8*, and *IL1B*, whereas PE addition reversed (*P* < 0.05) these changes and increased (*P* < 0.05) ileal *IL10* expression. Supplemental PE moderated ileal microbiota dysbiosis in challenged birds, characterized by a reduced abundance of *Firmicutes* along with increased abundances of *Bacteroidetes* (*Bacteroides*), *Deferribacteres* and several butyrate-producers such as *Prevotellaceae*, *Faecalibacterium*, *Blautia*, *Butyricicoccus, Lachnoclostridium*, and *Olsenella*, which may assist with energy harvesting and boost anti-inflammatory capacity of host. The decreased abundance of *Firmicutes* with the increased abundance of *Bacteroidetes* caused by PE addition had positive correlations with the decreased expression of ileal pro-inflammatory cytokines. The increased abundances of *Bacteroidetes* (*Bacteroides*) and *Prevotellaceae* following PE addition were also positively correlated with the improvement of performance (egg production and feed intake) of laying hens. Collectively, supplemental PE from *Flos lonicerae* in combination with *Baikal skullcap* alleviated *S. pullorum*-induced intestinal disruption and performance impairment in laying hens, which could be at least partially responsible by the modulation of gut microbial composition.

## Introduction

*Salmonella pullorum* is a fowl-specific pathogen that is common in the poultry industry of China (Guo et al., 2018). *S. pullorum* challenge is responsible for considerable disorders such as intestinal inflammation and barrier dysfunction as well as compromised production performance of chickens (Wang et al., 2012; Wu Q.J. et al., 2018). Due to the increasing emergence of resistant bacteria and antibiotic residues in animal products, a stipulation had been drawn for the prohibition of antibiotics used in the feed of layers during the laying period, resulting in a necessity to seek for the available countermeasures to control or limit the adverse effects of *S. pullorum* challenge in laying hens. Recent studies have focused on the importance of plant extracts (PE) as potential approaches to alleviate bacteria-related intestinal dysfunction and performance compromise in broiler chickens (Bozkurt et al., 2016; Abudabos et al., 2017). However, little information was available regarding whether dietary addition of certain PE could alleviate *S. pullorum*-induced intestinal disruption and performance impairment in laying hens.

*Flos lonicerae* and *Baikal skullcap* are widely planted in China and used as the sources of traditional herbal medicines (Zhou et al., 2017). The extracts from *Flos lonicerae* and *Baikal skullcap* were associated with various biological properties such as heat-clearing, antioxidation and immune-regulation (Mo et al., 2012). The principal active components of *Flos lonicerae* and *Baikal skullcap* are respectively characterized as chlorogenic acid and baicalin (Kaplya et al., 2004), which were associated with antibacterial and anti-inflammation properties *in vitro* (Lou et al., 2011; Palocz et al., 2016; Fu et al., 2018; Wu S.C. et al., 2018). It was also reported that chlorogenic acid or baicalin addition modulated immune responses and improved intestinal homeostasis in pigs and mice (Chen et al., 2018b; Wu S.C. et al., 2018). A combination of the supplementation of chlorogenic acid and geniposide (a kind of glycoside that similar to baicalin in structure) was demonstrated to mitigate high-fat diet-induced intestinal inflammation and barrier dysfunction in mice (Peng et al., 2018). Accordingly, addition of the extracts from *Flos lonicerae* and *Baikal skullcap* may exert active roles in protecting the intestinal tract in chickens. The current study was aimed to verify the potential alleviation of them for *S. pullorum*-induced intestinal disruption in laying hens.

*Salmonella* challenge was suggested to result in disturbance of gut microbiota in chickens (Li et al., 2017), which plays a functional role in the course of bacterial infection and contributes to maintenance of multiple physiological processes of host (Ferreira et al., 2011; Susan et al., 2014). There was an evidence that the improvements in growth performance and intestinal health in chickens could be established by the modulation of gut microbiota (Crisol-Martínez et al., 2017), which helped to attenuate intestinal inflammation and recover intestinal mucosa from injury (Wang et al., 2016; Crisol-Martínez et al., 2017). Hence, it is necessary to decipher gut microbial composition in order to better understand the potential influence of its manipulation by dietary intervention on the production performance and intestinal health of host. Growing findings supported the important roles of gut microbiota in influencing the bioactivity and bioavailability of plant-derived products such as flavonoids and polyphenols (Ozdal et al., 2016). A modulation of gut microbiome in broilers was confirmed as a consequence of dietary addition with a certain kind of PE (Huang et al., 2018). However, it is largely unknown whether PE addition can moderate gut microbiota dysbiosis, thereby conducing to the protection of intestine against bacterial challenge in laying hens. In keeping with this, the present study was conducted to assess the remodeling of gut microbiota to explain the possible alleviation of *S. pullorum*-induced intestinal disruption of laying hens by the supplementation of PE from *Flos lonicerae* in combination with *Baikal skullcap*.

## Materials and Methods

### Animals and Experimental Design

The experimental animal protocol for this study was approved by the Animal Care and Use Committee of the Feed Research Institute of Chinese Academy of Agricultural Sciences. A total of 216 41-weeks-old Jinghong laying hens were randomly allocated into 3 groups, with 6 replicates of 12 birds each. Initial body weight and egg production were similar across all the replicates. All birds were acclimated to a basal diet and environment for 1 week. The treatment groups were as follows: negative control (NC) group, birds were fed a basal diet and free of challenge; positive control (PC) group, birds were fed a basal diet and challenged with *S. pullorum*; treatment (T) group, birds were fed a basal diet supplemented with 1000 mg/kg PE and challenged with *S. pullorum*. The PE was consisted by *Flos lonicerae* extract (containing 10% chlorogenic acid) and *Baikal skullcap* extract (containing 90% baicalin) at a ratio of 3:2 (the final concentrations of chlorogenic acid and baicalin were 60 and 360 mg/kg, respectively), enabling this product to function optimally on the basis of a preliminary unpublished work. The product was provided by Centre Technology Co., Ltd. (Beijing, China). All birds were housed in three-tier battery cages and exposed to 16 h of light/day. Room temperature was maintained between 18 and 22°C throughout the experiment. All birds were allowed free access to feed and water. The composition of basal diet based on National Research Council (National Research Council, 1994) is shown in Table 1. Egg weight and egg number were recorded daily from each replicate.

**TABLE 1:** Composition of the basal diet (air-dry basis).

**Ingredients**	**Contents (g/kg of diet)**
Corn	654
Soybean meal	236
Limestone	89.3
Sodium chloride	3.0
Dicalcium phosphate	13.5
Choline chloride (50%)	1.0
DL-Methionine (98%)	1.0
Multimineral^1^	2.0
Multivitamin^2^	0.2
Nutrient levels	
Metabolizable energy (MJ/kg)	11.16
Crude protein	165.0
Total phosphorus	5.4
Available phosphorus	3.3
Calcium	33.1
Lysine	8.6
Methionine	3.7

*^1^Supplied per kilogram of diet: Cu, 8 mg; Zn, 66 mg; Fe, 60 mg; Mn, 65 mg; Se, 0.3 mg; I, 1 mg. ^2^Supplied per kilogram of diet: vitamin A, 12,500 IU; vitamin D_3_, 4,125 IU; vitamin E, 15 IU; menadione, 2 mg; thiamin, 1 mg; riboflavin, 8.5 mg; pyridoxine, 8 mg; cobalamin, 5 mg; pantothenic acid, 50 mg; niacin, 32.5 mg; biotin, 2 mg; folic acid, 5 mg; choline, 500 mg.*

### Oral Challenge and Sampling

The *S. pullorum* strain (CVCC533, China Veterinary Culture Collection Center, Beijing, China) was cultured in lactose broth at 37°C for 16 h. To enumerate the bacteria, inoculum was diluted and plated on *Salmonella*-*Shigella* agar (Hopebio Biotechnology Co., Ltd., Qingdao, China) at 37°C for 24 h. At the end of week 4 of the experiment period, all birds except those in NC were orally gavaged with 1 mL of *S. pullorum* culture (1.0 × 10^9^ CFU/mL) or the same amount of lactose broth. One bird per replicate was randomly selected for sample collection at 3 and 28 days post-infection (DPI). Individual blood were taken aseptically from the wing vein, serum samples were separated by centrifugation of blood at 3,000 rpm for 10 min at 4°C and stored at –20°C until analyzed. After blood collection, these birds were slaughtered rapidly, a little patch of heart, liver, and lung of each bird were harvested and preserved in –20°C for detecting the percentage of *Salmonella-*positive organs. Besides, the midpoint of ileal segment and ileal digesta of each bird were obtained and quick-freezed using liquid nitrogen, followed by the storage at −80°C until further analysis.

### Performance Measurement

Total feed intake was recorded for each replicate at the end of week 4 and 8 of the experiment. Egg production (calculated as an average hen-day production), egg weight, average daily feed intake (ADFI) and feed conversion ratio (FCR, defined as the ratio of feed intake to egg mass) were calculated out based on the periods of week 1–4, week 5–8, and week 1–8 of the experiment.

### Egg Quality Determination

At the end of week 4 and 8 of the experiment, 4 eggs per replicate were randomly selected for the determination of egg quality. Eggshell-breaking strength was measured using an Egg Force Reader (Orka Technology Ltd., Ramat Hasharon, Israel). Eggshell thickness was expressed as the mean value of measurements obtained at 3 locations on the surface of the egg (i.e., the air cell, equator, and sharp end) using an Eggshell Thickness Gauge (ESTG1, Orka Technology Ltd., Ramat Hasharon, Israel). Albumen height, Haugh unit, and yolk color were determined with an Egg Analyzer (Orka Technology Ltd., Ramat Hasharon, Israel).

### Serum Sample Analysis

Serum immunoglobulins (Ig) including IgY, IgA, and IgM were quantified separately with the commercial chicken-specific ELISA kits (Enzyme-linked Biotechnology Co., Ltd., Shanghai, China). All the procedures were carried out according to the manufacturers’ instructions. The intra-assay precision and inter-assay precision (variable coefficient) were less than 8 and 10%, respectively. Serum endotoxin concentration was measured using a chromogenic substrate assay kit (Jiancheng Bioengineering Institute, Nanjing, China). Briefly, 0.1 mL serum was incubated with 0.1 ml Limulus amebocyte lysate at 37°C for 45 min. After several subsequent reactions, the samples were read spectrophotometrically at 545 nm. The endotoxin level was calculated based on a standard curve of endotoxin concentrations.

### Bacteriological Analysis of Visceral Organs

The organ invasion after oral *S. pullorum* inoculation was measured as described previously (Shao et al., 2013). Briefly, samples of visceral organs including liver, heart and lung were weighed and homogenized separately with phosphate buffered solution (PBS). The homogenates of each organ were diluted 1:10 with a sterile solution of PBS and pre-enriched for 24 h at 37°C in lactose broth (Hopebio Biotechnology Co., Ltd., Qingdao, China). After this incubation, 100 μL of suspension was spread on *Salmonella*-*Shigella* agar (Hopebio Biotechnology Co., Ltd., Qingdao, China) for 24 h at 37°C, followed by the observation for the presence or absence of characteristic *Salmonella* colonies. Organ invasion rate was calculated by the ratio of *Salmonella*-positive samples to the total number of samples.

### RNA Isolation and Real-Time Quantitative PCR

Total RNA was extracted from the ileum by using Trizol Reagent (TIANGEN Biotech. Co., Ltd., Beijing, China) following the manufacturer’s protocols. Extracted RNA was dissolved in RNase-free water and quantified using an UV/Visible spectrophotometer (Amersham Bioscience, Sweden) at an absorbance of 260 nm. The quality of RNA was estimated from the absorbance ratio at 260 to 280 nm and by determination of the 18S and 28S bands after electrophoresis in 1% agarose gels stained with ethidium bromide. The cDNA samples were obtained by reverse transcription of total RNA using TIANGEN QuantScript RT kit (TIANGEN Biotech. Co., Ltd., Beijing, China). Real-time PCR for measuring ileal gene expression was carried out using RealMasterMix-SYBR Green kit (TIANGEN Biotech. Co., Ltd., Beijing, China) in an iCycler iQ5 multicolor real-time PCR detection system (Bio-Rad Laboratories, Hercules, CA, United States). The expression of *ACTB* (β-actin) was used as an internal control to normalize the amount of initial RNA for each sample. The primer sequences for the target genes (*IL1B*, interleukin 1beta; *IL-8*, interleukin 8; *TNFA*, tumor necrosis factor-alpha; *IFNG*, interferon-gamma; *IL10*, interleukin 10) and reference gene are shown in Table 2. The protocol for all the genes was as follows: 95°C for 5 min; 40 cycles of 95°C for 10 s, 60°C for 30 s. All measurements were carried out in duplicate. Amplification efficiency of each gene was validated by constructing a standard curve through serial dilutions of cDNA. Specificity of PCR products was evaluated by the analysis of melting curve. The results of relative mRNA expression of genes were calculated using the 2^–ΔΔCt^ method (Livak and Schmittgen, 2001).

**TABLE 2:** Sequences for real-time PCR primers.

**Genes^1^**	**Primer sequence^2^(5′–3′)**	**Accession no.**
*ACTB*	F: ATGATATTGCTGCGCTCGTT	L08165
	R: TCTTTCTGGCCCATACCAACC	
*IL1B*	F: ACTGGGCATCAAGGGCTACA	Y15006.1
	R: GCTGTCCAGGCGGTAGAAGA	
*IL8*	F: GGCTTGCTAGGGGAAATGA	DQ393272.2
	R: AGCTGACTCTGACTAGGAAACTGT	
*TNFA*	F: GCCCTTCCTGTAACCAGATG	GU230788.1
	R: ACACGACAGCCAAGTCAACG	
*IFNG*	F: GAACTGGACAGGGAGAAATGAGA	NM_205149.1
	R: ACGCCATCAGGAAGGTTGTT	
*IL10*	F: CATGCTGCTGGGCCTGAA	AJ621614
	R: CGTCTCCTTGATCTGCTTGATG	

*^1^*ACTB*, β-actin; *IL1B*, interleukin 1beta; *IL8*, interleukin 8; *TNFA*, tumor necrosis factor-alpha; *IFNG*, interferon-gamma; *IL10*, interleukin 10. ^2^F, forward; R, reverse.*

### Sequencing of Ileal Microbiota

Microbial DNA was extracted from the ileal content using NucleoSpin^®^ DNA Stool kit (Macherey-Nagel company, Germany). The quality of extracted DNA were checked with gel electrophoresis. Bacterial 16S rDNA sequences spanning the variable regions V3–V4 were amplified using primer 338F (5′-ACT CCT ACG GGA GGC AGC A-3′) and 806R (5′-GGA CTA CHV GGG TWT CTA AT-3′). Amplification by PCR consisted of the following procedures: denaturation at 95°C for 5 min, twenty-five cycles of 30 s at 95°C, 50°C for 30 s, and 72°C for 40 s, with a final extension at 72°C for 7 min. High-throughput sequencing of PCR products was carried out on the Illumina HiSeq2500 PE250 platform (Illumina, San Diego, United States) at Biomarker Technology Co., Ltd. (Beijing, China). The sequencing results has been submitted to the Sequence Read Archive of the NCBI (accession number: PRJNA535413). The raw paired-end reads from the original DNA fragments were merged using FLASH v1.2.7 (Magoc and Salzberg, 2011). All of the effective reads from each sample were clustered into operational taxonomic units (OTUs) based on a 97% sequence similarity identified by UCLUST in QIIME v1.8.0 (Edgar, 2010). Taxonomic classification at different taxonomic levels of OTU sequences were performed by comparing sequences to the GreenGene v13.8 database (DeSantis et al., 2006). Shannon and Simpson indices, Chao1 and ACE estimators were included in α-diversity analysis by using the MOTHUR v1.31.2 (Schloss et al., 2009). The principal coordinates analysis (PCoA) and partial least squares discriminant analysis (PLS-DA) plots based on unweighted Unifrac were used to estimate pairwise distances among samples and to establish β-diversity. Linear discriminant analysis (LDA) combined effect size measurements (LEfSe) and non-parametric *t*-test (with Metastats software) were employed to identify the biological differences among groups (White et al., 2009). Metagenome functional content from 16S rDNA was predicted using PICRUSt (Langille et al., 2013), based on KEGG and COG databases. Spearman correlation analysis was performed for the correlations between ileal cytokine expression and microbial composition as well as between performance parameters (feed efficiency, egg production and ADFI) and microbial composition.

### Data Processing and Analysis

Data are presented as mean ± standard deviation and analyzed by one-way ANOVA using the general linear model procedure of SPSS 18.0 software. Differences among different groups were analyzed by Duncan’s multiple comparisons. Significance was set at *P* < 0.05 and 0.05 < *P* < 0.10 was considered as a trend toward significance. Data set on the percentages of *Salmonella*-positive organs were subjected to Chi-Square test.

## Results

### Production Performance and Egg Quality

*Salmonella pullorum* challenge resulted in reductions (*P* < 0.05) in egg production and ADFI coupled with an increase (*P* < 0.05) in FCR of laying hens from week 5 to 8 and week 1 to 8 of the experiment (Table 3). However, birds in PE group had greater (*P* < 0.05) egg production and ADFI from week 5 to 8 and week 1 to 8 of the experiment, which was concomitant with a lower (*P* < 0.05) FCR from week 5 to 8 than those in PC group. No alterations (*P* > 0.05) in egg quality at week 4 of the experiment were noted among groups. There was little change in egg quality except for a reduction (*P* < 0.05) of eggshell strength at week 8 of the experiment in PC group as compared with NC group. However, birds in T group showed an enhanced (*P* < 0.05) eggshell strength at week 8 of the experiment in comparison with those in PC group.

**TABLE 3:** Effects of plant extracts from *Flos Lonicerae* in combination with *Baikal Skullcap* on production performance^1^ and egg quality of laying hens.

	**Time**	**NC^2^**	**PC**	**T**	***P*-value**
Egg production (%)	week 1–4	83.90 ± 5.16	83.94 ± 3.77	85.71 ± 4.15	0.724
	week 5–8	84.70 ± 0.74^a^	62.37 ± 0.44^c^	73.67 ± 1.24^b^	< 0.001
	week 1–8	84.30 ± 2.83^a^	73.15 ± 1.96^c^	79.49 ± 2.34^b^	< 0.001
Egg weight (g)	week 1–4	64.87 ± 0.55	64.82 ± 1.78	64.94 ± 1.94	0.992
	week 5–8	66.04 ± 1.37	64.21 ± 1.81	64.41 ± 1.21	0.094
	week 1–8	65.46 ± 0.95	64.52 ± 1.57	64.67 ± 1.54	0.469
ADFI (g)	week 1–4	117.65 ± 4.32	117.65 ± 4.32	117.68 ± 5.42	0.984
	week 5–8	117.88 ± 1.48^a^	93.18 ± 3.56^c^	105.26 ± 3.09^b^	< 0.001
	week 1–8	117.77 ± 1.62^a^	105.66 ± 3.04^c^	111.47 ± 2.40^b^	< 0.001
FCR	week 1–4	2.25 ± 0.15	2.21 ± 0.07	2.23 ± 0.06	0.918
	week 5–8	2.21 ± 0.06^c^	2.57 ± 0.20^a^	2.51 ± 0.16^b^	0.002
	week 1–8	2.22 ± 0.09^b^	2.39 ± 0.11^a^	2.36 ± 0.11^a^	0.025
Albumen height (mm)	week 4	6.37 ± 0.34	6.47 ± 0.43	6.91 ± 1.14	0.425
	week 8	7.11 ± 0.92	6.43 ± 0.75	6.89 ± 0.88	0.339
Haugh unit	week 4	77.26 ± 5.65	78.31 ± 6.16	79.28 ± 3.12	0.438
	week 8	81.99 ± 2.48	78.01 ± 2.80	79.63 ± 2.68	0.424
Yolk color	week 4	5.53 ± 0.37	6.00 ± 0.22	6.02 ± 1.01	0.295
	week 8	5.83 ± 0.54	6.10 ± 0.45	6.34 ± 0.62	0.272
Eggshell strength (N/m^2^)	week 4	33.50 ± 4.10	33.01 ± 1.89	36.41 ± 7.96	0.972
	week 8	35.13 ± 1.14^b^	32.01 ± 0.97^a^	34.80 ± 2.58^b^	0.003
Eggshell thickness (mm)	week 4	0.423 ± 0.015	0.434 ± 0.014	0.437 ± 0.004	0.167
	week 8	0.432 ± 0.008	0.422 ± 0.008	0.430 ± 0.010	0.130

*^*a*,*b*,*c*^Values with different superscripts within the same row differ significantly (*P* < 0.05). ^1^ADFI, average daily feed intake; FCR, feed conversion ratio. ^2^NC, negative control (birds were free of challenge); PC, positive control (birds were challenged with *S. pullorum* at the end of week 4 of the experiment); T, treatment group (PC + plant extracts addition at 1000 mg/kg).*

### Serum Biochemical Indices

Birds in T group had an increasing trend (*P* = 0.051) of serum IgY level with an elevated (*P* < 0.05) serum IgM level at 3 DPI than those in PC group (Table 4). In addition, serum IgY level at 28 DPI was increased in T group as compared to that in either NC or PC group. The *S. pullorum*-induced increase (*P* < 0.05) in serum endotoxin content at 3 DPI was reversed (*P* < 0.05) by PE treatment.

**TABLE 4:** Effects of plant extracts from *Flos Lonicerae* in combination with *Baikal Skullcap* on serum indices^1^ of laying hens at 3 and 28 days post *S. pullorum* infection.

	**Time**	**NC^2^**	**PC**	**T**	***P*-value**
IgY (mg/mL)	3 days	2.70 ± 0.86	1.95 ± 0.85	3.28 ± 0.86	0.051
	28 days	2.56 ± 0.69^b^	1.83 ± 0.66^b^	3.71 ± 0.95^a^	0.003
IgA (mg/mL)	3 days	0.50 ± 0.04	0.52 ± 0.12	0.61 ± 0.13	0.168
	28 days	0.53 ± 0.10	0.47 ± 0.13	0.63 ± 0.88	0.052
IgM (mg/mL)	3 days	0.72 ± 0.24^b^	0.79 ± 0.26^b^	1.29 ± 0.23^a^	0.002
	28 days	1.06 ± 0.37	0.72 ± 0.38	1.46 ± 0.76	0.087
Endotoxin (EU/mL)	3 days	0.50 ± 0.10^b^	0.78 ± 0.13^a^	0.52 ± 0.08^b^	0.001
	28 days	0.48 ± 0.16	0.68 ± 0.12	0.52 ± 0.15	0.067

*^*a*,*b*^Values with different superscripts within the same row differ significantly (*P* < 0.05). ^1^Ig, immunoglobulin. ^2^NC, negative control (birds were free of challenge); PC, positive control (birds were challenged with *S. pullorum* at the end of week 4 of the experiment); T, treatment group (PC + plant extracts addition at 1000 mg/kg).*

### Organ Invasion by *Salmonella*

The visceral organs including liver, heart and lung from NC group were all free of *Salmonella* after enrichment culture (Table 5), while 83.3% of the liver sample and 66.7% of the lung and heart samples taken in PC group at 3 DPI were positive for *Salmonella*. Birds in T group had lower (*P* < 0.05) percentage of *Salmonella*-positive liver sample at 3 DPI over that of PC group.

**TABLE 5:** Effects of plant extracts from *Flos Lonicerae* in combination with *Baikal Skullcap* on the percentage (%) of *Salmonella*-positive organs of laying hens at 3 days post *S. pullorum* infection.

	**NC^1^**	**PC**	**T**	***P*-value**
Liver	0^b^	83.3^a^	16.7^b^	0.025
Lung	0	66.7	50	0.095
Heart	0	66.7	50	0.095

*^*a*,*b*^Values with different superscripts within the same row differ significantly (*P* < 0.05). ^1^NC, negative control (birds were free of challenge); PC, positive control (birds were challenged with *S. pullorum* at the end of week 4 of the experiment); T, treatment group (PC + plant extracts addition at 1000 mg/kg).*

### Relative mRNA Expression of Ileal Cytokines

**FIGURE 1:**
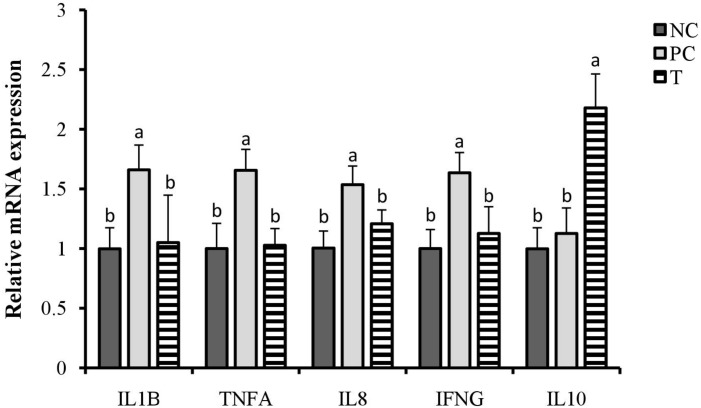
Effects of plant extracts (PE) from *Flos Lonicerae* in combination with *Baikal Skullcap* on the relative expression of ileal cytokines of laying hens at 3 days post *S. pullorum* infection. Error bars represent the standard deviation. ^*a,b*^Treatments with different letters were significantly different (*P* < 0.05). NC, negative control (birds were free of challenge); PC, positive control (birds were challenged with *S. pullorum* at the end of week 4 of the experiment); T, treatment group (PC + plant extracts addition at 1000 mg/kg).

There were increases (*P* < 0.05) in the relative expression of ileal *IFNG*, *TNFA*, *IL8*, and *IL1B* at 3 DPI of layers in response to *S. pullorum* challenge (Figure 1). However, they were all decreased (*P* < 0.05) in layers of T group when compared with those in PC group. Furthermore, an up-regulation (*P* < 0.05) in the relative expression of ileal *IL10* at 3 DPI was observed in the birds from T group relative to PC group.

### Diversity of Ileal Microbiota

**FIGURE 2:**
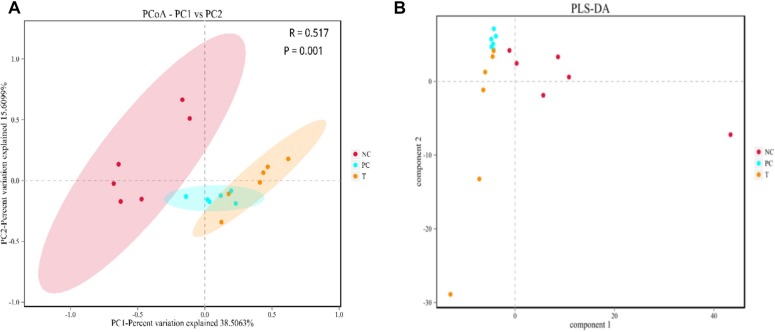
Beta-diversity analysis of ileal microbiota among groups at 3 days post *S. pullorum* infection. **(A)** principal co-ordinates analysis (PCoA). **(B)** Partial least squares discriminant analysis (PLS-DA). NC, negative control (birds were free of challenge); PC, positive control (birds were challenged with *S. pullorum* at the end of week 4 of the experiment); T, treatment group (PC + plant extracts addition at 1000 mg/kg).

The α-diversity including ACE and Chao1 estimators of ileal microbiota at 3 DPI were lower (*P* < 0.05) in the birds from PC group as compared with NC group (Supplementary Table S1). No change (*P* > 0.05) occurred in the α-diversity of ileal microbiota in response to PE treatment. To evaluate the similarity (β-diversity) of microbial community structure among groups, PCoA and PLS-DA were performed. PCoA plot showed a trend of separation of microbial communities at 3 DPI among groups (Figure 2A). PLS-DA plot also defined groups where the samples from different groups occupied distinct positions (Figure 2B).

### Composition of Ileal Microbiota

**FIGURE 3:**
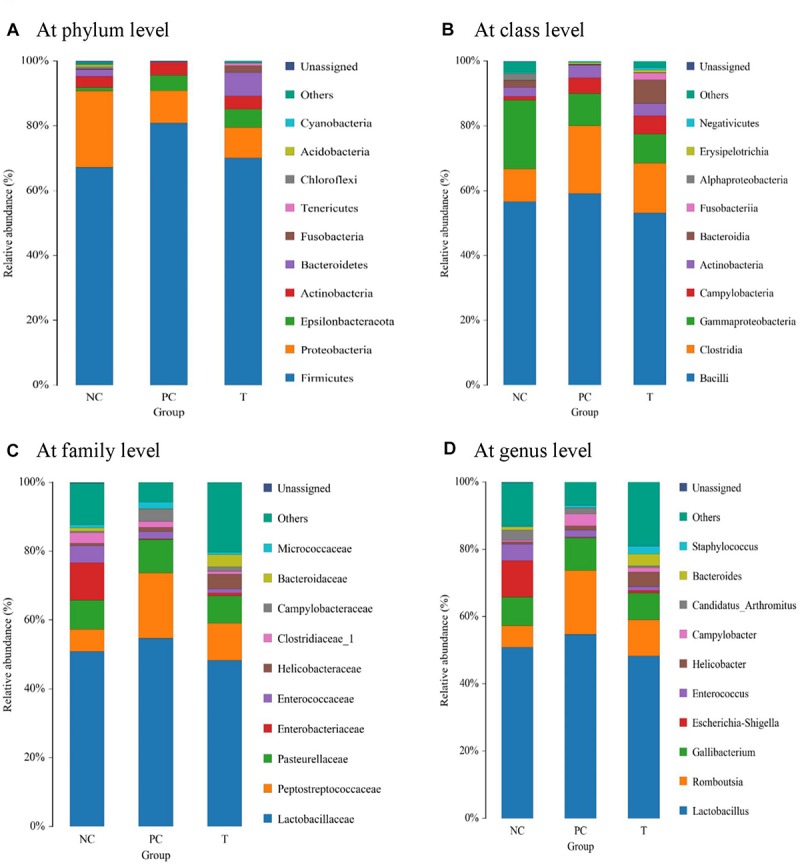
Analysis of ileal microbial composition at different taxonomic levels (**A** at phylum level, **B** at class level, **C** at family level, and **D** at genus level) of laying hens at 3 days post *S. pullorum* infection. NC, negative control (birds were free of challenge); PC, positive control (birds were challenged with *S. pullorum* at the end of week 4 of the experiment); T, treatment group (PC + plant extracts addition at 1000 mg/kg).

The dominant phyla across all the groups were *Firmicutes* and *Proteobacteria*, which together contributed greater than 80% of the whole phyla (Figure 3A). *S. pullorum* challenge led to a reduced abundance of *Proteobacteria* with an increased abundance of *Firmicutes* at 3 DPI, when PE addition reduced the abundance of *Firmicutes* and increased *Bacteroidetes* abundance. Within *Firmicutes*, the majority belonged to the classes *Bacilli*, *Clostridia* and *Gammaproteobacteria* (Figure 3B). Family level analysis showed that the microbiota of layers was dominated by *Lactobacillaceae*, *Peptostreptococcaceae*, and *Pasteurellaceae* (Figure 3C). The increased abundance of *Peptostreptococcaceae* at 3 DPI in challenged birds was relieved by PE addition, which also increased the abundance of *Bacteroidaceae*. At genus level, the *Lactobacillus* and *Romboutsia* accounted for the largest proportion of the microbiota (Figure 3D). The abundance of *Romboutsia* at 3 DPI was increased in challenged birds but diminished by PE addition, which also induced an increase in *Bacteroides* abundance compared to PC. The averages of the relative abundance of bacterial groups at various taxonomic levels of layers at 3 DPI were exhibited in Supplementary Table S2.

**FIGURE 4:**
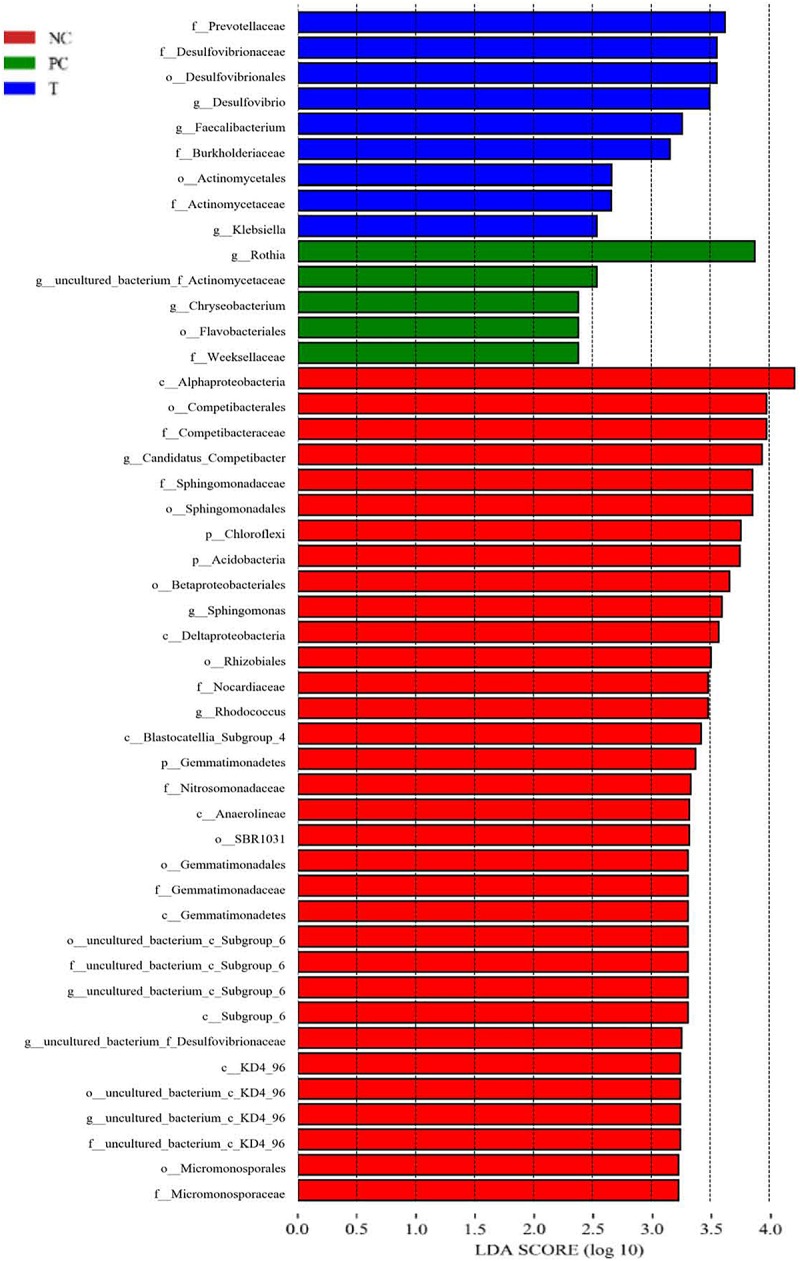
Linear discriminant analysis (LDA) combined effect size measurements (LEfSe) analysis of ileal microbiota in laying hens at 3 days post *S. pullorum* infection. Species with significant difference that have an LDA score greater than the estimated value (2.0). The length of the histogram represents the LDA score. NC, negative control (birds were free of challenge); PC, positive control (birds were challenged with *S. pullorum* at the end of week 4 of the experiment); T, treatment group (PC + plant extracts addition at 1000 mg/kg).

LEfSe analysis was applied to identify the relative richness (*P* < 0.05, LDA > 2.0) of bacterial members in groups (Figure 4). There were substantial members enriched in the ileum of NC birds at 3 DPI. *Rothia* was found to be enriched in birds of PC group, whereas the microbiota from T group was differentially enriched with *Prevotellaceae*, *Desulfovibrionaceae* (*Desulfovibrio*), *Burkholderiaceae* and *Faecalibacterium*. Non-parametric *t*-test was further employed to explore the differences in the microbial composition (Table 6). *S. pullorum* challenge reduced (*P* < 0.05) the abundances of *Deferribacteres*, *Ruminococcus torques*, *Olsenella* and *Subdoligranulum* at 3 DPI, and simultaneously tended to decrease (*P* < 0.10) the abundances of *R. gauvreauii*, *Blautia*, *Butyricicoccus*, *Lachnoclostridium* and *Mesorhizobium*. *Bacillus* and *Rothia* at 3 DPI appeared at higher (*P* < 0.05) abundances in PC group than those in NC group. There were elevations (*P* < 0.05) in the abundances of *Blautia* and *Butyricicoccus* with increasing trends (*P* < 0.10) of the abundances of *Deferribacteres*, *R. gauvreauii*, *R. torques*, *Lachnoclostridium*, *Olsenella* and *Subdoligranulum* at 3 DPI in response to PE addition, which also decreased (*P* < 0.05) the abundances of *Bacillus* and *Rothia*.

**TABLE 6:** Differential species^1^ (%) identified from the ileal microbiota of laying hens from different groups.

	**NC^2^**	**PC**	**T**	***P*-values**	
				**NC vs. PC**	**PC vs. T**
Phylum					
*Deferribacteres*	0.140 ± 0.134	0.001 ± 0.001	0.007 ± 0.003	0.035	0.067
Genera					
*R. gauvreauii_*	0.0053 ± 0.0032	0.0009 ± 0.0006	0.0196 ± 0.0136	0.092	0.099
*R. torques*	0.0436 ± 0.0208	0.0039 ± 0.0015	0.0294 ± 0.0208	0.012	0.096
*Bacillus*	0.0057 ± 0.0024	0.0290 ± 0.0102	0.0043 ± 0.0022	0.005	0.007
*Blautia*	0.0195 ± 0.0137	0.0009 ± 0.0006	0.0089 ± 0.0039	0.098	0.017
*Butyricicoccus*	0.0097 ± 0.0060	0.0007 ± 0.0007	0.0321 ± 0.0170	0.062	0.027
*Lachnoclostridium*	0.045 ± 0.027	0.006 ± 0.001	0.032 ± 0.022	0.079	0.091
*Mesorhizobium*	0.0711 ± 0.0498	0.0021 ± 0.0011	0.0003 ± 0.0003	0.091	0.056
*Olsenella*	0.032 ± 0.014	0.002 ± 0.001	0.016 ± 0.011	0.007	0.077
*Rothia*	0.40 ± 0.24	1.70 ± 0.79	0.16 ± 0.07	0.043	0.021
*Subdoligranulum*	0.041 ± 0.021	0.002 ± 0.001	0.013 ± 0.009	0.012	0.096

*^1^*R. gauvreauii*, *Ruminococcus gauvreauii*; *R. torques*, *Ruminococcus torques*. ^2^NC, negative control (birds were free of challenge); PC, positive control (birds were challenged with *S. pullorum* at the end of week 4 of the experiment); T, treatment group (PC + plant extracts addition at 1000 mg/kg).*

### Functional Prediction of Ileal Microbiota

Presumptive functions of ileal microbiota of layers at 3 DPI were examined by using PICRUSt. According to the KEGG prediction (Supplementary Figure S1), the microbiota represented in the ileum was mainly associated with the following biological processes: amino acid metabolism, carbohydrate metabolism, energy metabolism and nucleotide metabolism. Metagenomic prediction based on COG categories revealed that the functional pathways enriched within the microbiota were those corresponding to general function prediction only, amino acid transport and metabolism, carbohydrate transport and metabolism, energy production and conversion as well as transcription. No differences (*P* > 0.10) were noted in the predicted pathways of the microbiota among groups.

### Correlation Between Ileal Microbiota and Ileal Inflammation

**FIGURE 5:**
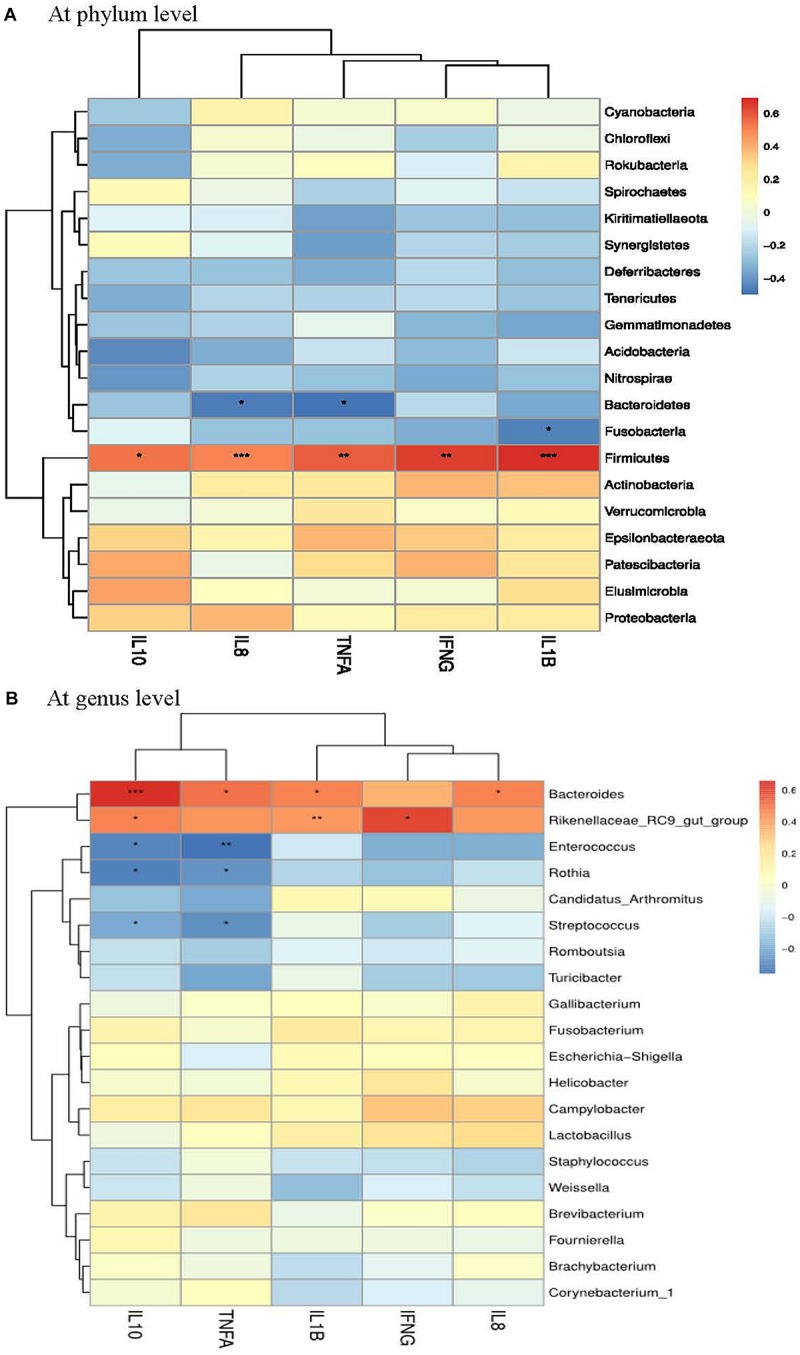
Correlation analysis between ileal cytokine expression and ileal microbial composition (**A** at phyum level and **B** at genus level) in laying hens at 3 days post *S. pullorum* infection. *IL1B*, interleukin 1beta; *IL8*, interleukin 8; *TNFA*, tumor necrosis factor-alpha; *IFNG*, interferon-gamma; *IL10*, interleukin 10. The red and blue panes, respectively, represent positive and negative correlations between ileal cytokine expression and microbiota (color intensity shows the Spearman’s *r*-value of correlation in each panel). The asterisks indicate significant correlations at *P* < 0.05 level (^*^P < 0.05; ^∗∗^*P* < 0.01; ^∗∗∗^*P* < 0.001).

A Spearman’s rank correlation analysis was employed to identify specific bacterial members associated with the differential expression of ileal cytokines among groups. The abundance of phylum *Firmicutes* showed highly positive correlations (*P* < 0.01) with the expression of *IFNG*, *IL1B, TNFA* and *IL8* (Figure 5A). In contrast, the abundance of phylum *Bacteroidetes* was negatively correlated (*P* < 0.05) with *TNFA* and *IL8* expression. The abundance of genus *Bacteroides* was positively correlated (*P* < 0.05) with *IL1B, TNFA* and *IL8* expression (Figure 5B), but it also had a highly positive correlation (*P* < 0.01) with *IL10* expression. Besides, *Rothia* abundance was negatively correlated (*P* < 0.05) with the expression of both *TNFA* and *IL10*.

### Correlation Between Ileal Microbiota and Production Performance

**FIGURE 6:**
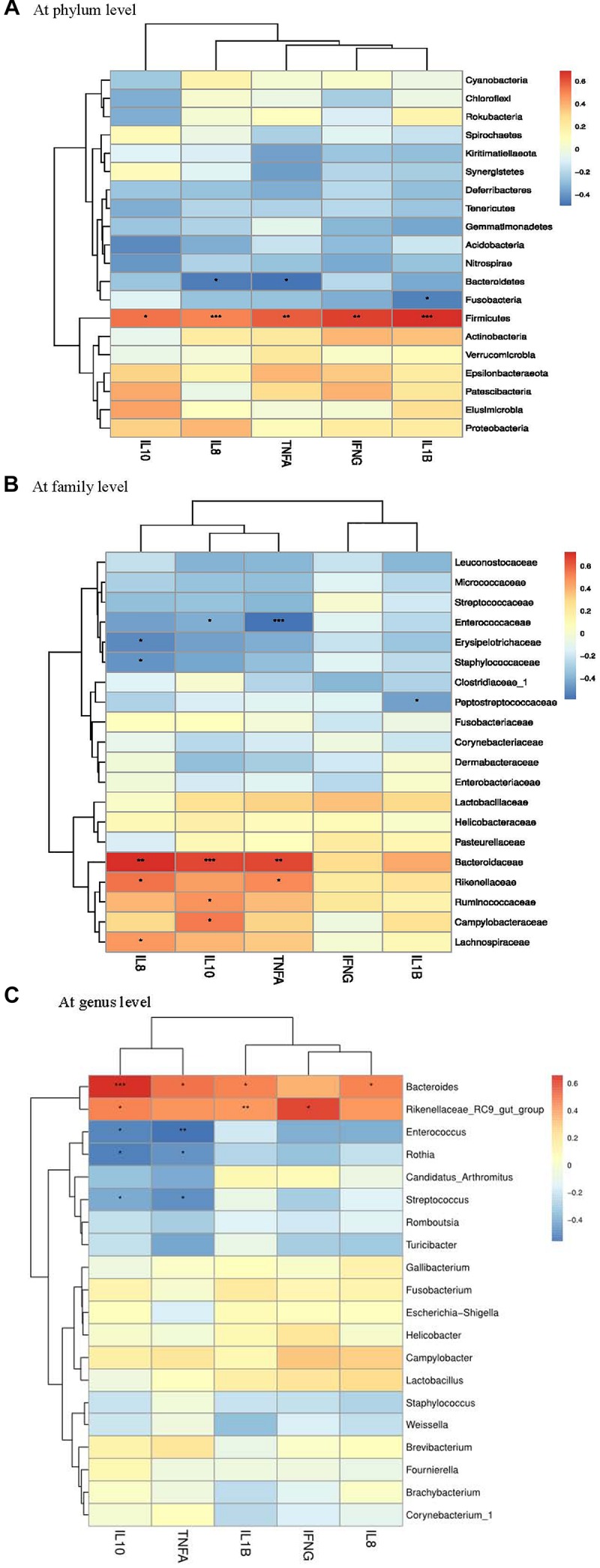
Correlation analysis between performance parameters and ileal microbial composition (**A** at phylum level, **B** at family level, and **C** at genus level) in laying hens at 3 days post *S. pullorum* infection. FE, feed efficiency; LR, laying rate (egg production); ADFI, average daily feed intake. The red and blue panes, respectively, represent positive and negative correlations between performance parameters and microbiota (color intensity shows the (Spearman’s *r*-value of correlation in each panel). The asterisks indicate significant correlations at *P* < 0.05 level (^*^P < 0.05; ^∗∗^*P* < 0.01; ^∗∗∗^*P* < 0.001).)

A Spearman’s rank correlation analysis was performed to evaluate the potential association between alterations in ileal microbiota and performance parameters of laying hens. There was a positive correlation between the phylum *Deferribacteres* and ADFI (Figure 6A), while the phylum *Bacteroidetes* was positively correlated with egg production and ADFI. The families *Desulfovibrionaceae*, *Lachnospiraceae*, *Bacteroidaceae*, *Prevotellaceae* and *Ruminococcaceae* had positive correlations with both egg production and ADFI (Figure 6B). Moreover, positive correlations were detected between *Bacteroides* and performance parameters (feed efficiency, egg production and ADFI) (Figure 6C).

## Discussion

Previous studies evidenced that supplemental PE based on baicalin or chlorogenic acid was associated with an improvement in growth performance of animals (Chen et al., 2018a; Wu S.C. et al., 2018). Similarly, the current study revealed a mitigation of *S. pullorum*-induced impairment of production performance of laying hens in the presence of PE, indicated by the increased egg production and ADFI as well as feed efficiency. With regard to egg quality, the challenge resulted in a reduction of eggshell strength of layers. This might be derived from the *Salmonella*-induced compromised absorption of microelements such as manganese and zinc (Liu et al., 2012; Diaz-Ochoa et al., 2016), which are critical for eggshell formation (Gheisari et al., 2011). Thereby, the enhanced eggshell strength of layers might be the result of a restoration of *S. pullorum*-induced malabsorption of microelements following PE addition.

Immunoglobulins that produced by lymphocytes are critical for protecting against toxins and bacteria. Their levels are viewed as an important indicator of humoral immunity (Schroeder and Cavacini, 2010). In the current study, supplemental PE resulted in an increasing trend of serum IgY level with an elevated IgM level of birds at 3 DPI, indicating a reinforcement of humoral immunity that could be beneficial for the resistance of host to *S. pullorum* invasion. Endotoxin is part of the cell wall of gram-negative bacteria such as *Salmonella* and released into circulation when the intestinal barrier is destroyed. Serum endotoxin content can be served as an indicator for monitoring the extent of intestinal damage (Mani et al., 2012). Herein, an elevation in serum endotoxin content was observed in challenged birds, suggesting the idea of an impairment of gut barrier function as a result of *S. pullorum* challenge. In support of this view, translocations of *Salmonella* to the liver, lung and heart samples were observed following *S. pullorum* challenge. However, PE addition lowered serum endotoxin content and the frequency of *Salmonella*-positive liver sample in challenged birds, bringing an evidence for the attenuation of *S. pullorum*-induced intestinal disruption of laying hens following PE addition. Similar results were found in some previous studies (Chen et al., 2018b; Peng et al., 2018), in which supplemental baicalin or chlorogenic acid protected intestinal barrier against challenge in animals.

In concert with a previous study (Wei et al., 2018), the present study showed that *S. pullorum* challenge induced gut inflammation in laying hens, evidenced by the elevated expression of ileal pro-inflammatory cytokines inclusive of *TNFA*, *IFNG*, *IL1B*, and *IL8*. Although inflammatory responses play a crucial role in immune defense against infection (Withanage et al., 2005), the sharp increase of pro-inflammatory cytokines aggravated intestinal damage and induced high consumption of nutrients (Dinarello, 2000), which could subsequently transform into an impaired performance in animals (Pluske et al., 2018). Thereby, the induction of ileal inflammation mediated by pro-inflammatory cytokines conduced to the intestinal disruption and performance impairment in laying hens challenged with *S. pullorum*. However, there were reductions in the expression of ileal *TNFA*, *IFNG*, *IL1B*, and *IL8* in challenged birds received a diet containing PE, providing a clue for the role of PE addition in remitting *S. pullorum*-induced intestinal inflammation, which might subsequently favor the attenuation of the impaired performance of laying hens. This is in accordance with the responses to baicalin and chlorogenic acid treatments *in vitro* and in animals, where gut inflammation was attenuated (Palocz et al., 2016; Chen et al., 2018b; Fu et al., 2018; Wu S.C. et al., 2018). It is of interest to notify an up-regulated expression of ileal *IL10* in response to PE addition. *IL10* is a key anti-inflammatory cytokine secreted by macrophages that serves to maintain immune balance by inhibiting the excessive production of pro-inflammatory cytokines (Corwin, 2000). As a result, an up-regulated *IL10* expression could be associated with the alleviation of *S. pullorum*-induced ileal inflammation in laying hens fed with PE.

In attempts to better understand the origins of the mitigative effects of PE addition on the *S. pullorum*-induced intestinal disruption of layers, the focus of this study was on the analysis of gut microbiota, which could mediate the *Salmonella*-induced inflammatory environment of intestine in animals (Ferreira et al., 2011). Similar to a previous report (Price et al., 2010), the current study revealed that *S. pullorum* challenge induced a shift in microenvironment, favoring *Firmicutes* at the expense of *Proteobacteria* and *Bacteroidetes*, while PE addition increased the abundance of *Bacteroidetes* and reduced the abundance of *Firmicutes*. Increased *Firmicutes* and decreased *Bacteroidetes* were associated with gut inflammation and barrier dysfunction in animals (Rabot et al., 2016; Las-Heras et al., 2019). Besides, elevated *Bacteroidetes* conduced to the protection against *Salmonella*-induced gut inflammation (Ferreira et al., 2011). Analogously, this study showed that *Firmicutes* abundance had highly positive relationships with the expression of ileal pro-inflammatory cytokines (*IL1B*, *IL8*, *TNFA* and *IFNG*), while *Bacteroidetes* abundance was negatively correlated with the expression of *IL8* and *TNFA*. In addition, although the genus *Bacteroides* was positively correlated with the expression of *IL1B*, *IL8* and *TNFA*, it also had a highly positively correlation with *IL10* expression, which may assist with anti-inflammatory response. Thereby, the increased abundances of *Bacteroidetes* (*Bacteroides*) along with the reduced abundance of *Firmicutes* in the ileum might contribute to the attenuation of *S. pullorum*-induced gut inflammation of layers following PE addition.

Several species were identified as biomarkers to distinguish ileal microbiota of layers among groups, such as *Prevotellaceae*, *Desulfovibrionaceae*, *Burkholderiaceae* and *Faecalibacterium*. *Prevotellaceae* is known for the production of succinate that facilitates energy metabolism of host through tricarboxylic acid cycle (De-Vadder et al., 2016). *Prevotellaceae* was also associated with short-chain fatty acids (SCFAs) generation in gut because of its capability to produce various polysaccharidases to degrade cellulose and insoluble starch, which was essential for animals to be able to digest plant-based diets (Wang et al., 2018), resulting in a positive association between gut *Prevotellaceae* and feed efficiency in animals (Quan et al., 2019). *Faecalibacterium* in chicken gut represented an important bacterial group closely related to the production of butyrate (Bjerrum et al., 2006), which serves as intestinal epithelial specific nutrient and energy component, favoring the suppression of inflammatory responses and recovery of intestinal injury via multiple ways (Singh N. et al., 2014). *Desulfovibrionaceae* was showed to be implicated in propionic acid production that aids in energy harvest of host (Liu et al., 2019). The functional roles of *Burkholderiaceae* in animal gut remain unclear. However, an increase in gut *Burkholderia* was accompanied by the improvements of intestinal structure and growth performance in chickens following a probiotic addition (Li et al., 2019), implying a benefit of *Burkholderia* for gut health of host. To sum up, the ileal enrichments of *Prevotellaceae*, *Faecalibacterium* and *Burkholderiaceae* could conduce to the improved performance along with the attenuated ileal inflammation in challenged birds following PE addition.

Further analysis revealed more differential species at various taxonomic levels among groups. For example, there was a restoration of PE addition in *S. pullorum*-induced reduction of ileal *Deferribacteres* that is viewed as a kind of iron-reducer (Miroshnichenko et al., 2003). Iron ion is a nutrient that often limits the growth of bacteria such as *Salmonella*, and animals typically try to reduce biologically available iron in order to avoid bacterial infection (Deriu et al., 2013). It was thus speculated that the increased *Deferribacteres* following PE addition contributed to the restriction of *S. pullorum* invasion, evidenced by a reduction of *Salmonella*-positive organ in layers. *Blautia*, *Butyricicoccus*, and *Lachnoclostridium* were proposed as butyrate-producers that implicated in the alleviation of gut inflammation (Zhang X. et al., 2015; Takahashi et al., 2016; Zhao et al., 2017; Pérez-Burillo et al., 2019). *Ruminococcaceae* contains substantial cellulolytic-degrading bacteria such as *R. gauvreauii* and *R. torques*, whose reductions were related to the occurrence of gut inflammation due to the lessened SCFAs production (Domingo et al., 2008; Takahashi et al., 2016; Halfvarson et al., 2017). *Subdoligranulum* and *Olsenella* also had abilities to ferment polysaccharides to SCFAs such as acetate and butyrate (Zhang J. et al., 2015; Ricaboni et al., 2017; Li et al., 2018). Besides, *Subdoligranulum* was capable of producing bacteriocins that can confine *Salmonellla* growth (Yang et al., 2013). An increased abundance of gut *Subdoligranulum* was confirmed to be accompanied by an improved production performance in laying hens (Guo et al., 2017). *Rothia* was viewed as a harmful bacterium that associated with gut inflammation occurrence (Bajer et al., 2017). In this study, PE addition triggered increases in *Blautia*, *Butyricicoccus*, *Lachnoclostridium, R. gauvreauii*, *R. torques, Subdoligranulum*, and *Olsenella*, concurrent with a reduction of *Rothia*, suggesting the ileal microbiota of birds fed with PE becomes more efficient in protecting intestine against *S. pullorum*-induced inflammation and assisting in energy harvest of host.

Gut microbiota has profound impacts on the bioavailability of dietary components, playing prominent roles in host nutritional processes (Susan et al., 2014). It was thought that gut *Firmicutes* and *Bacteroidetes* were both closely related to chicken performance (Singh K.M. et al., 2014), due to their roles in polysaccharide decomposition and the subsequent generation of SCFAs that aid in host energy utilization (Postler and Ghosh, 2017). However, no influence was found of the change in the abundances of gut *Firmicutes* and *Bacteroidetes* on SCFAs production and energy harvest in mice (Murphy et al., 2010). Contradictorily, Singh K.M. et al. (2014) reported that broilers with high feed efficiency exhibited a higher abundance of *Bacteroides* in gut. Wang et al. (2019) observed an increased abundance of *Bacteroidetes* and a reduced abundance of *Firmicutes* accompanied by an improvement of growth performance in pigs. The above studies highlighted a complexity of the linkage between these two phyla and SCFAs-related energy harvest of host (Andoh et al., 2016). In this study, *Bacteroidetes* together with the families *Bacteroidaceae*, *Desulfovibrionaceae*, and *Prevotellaceae* elicited positive correlations with both egg production and ADFI. *Bacteroides* was also positively correlated with feed efficiency, egg production and ADFI. Thus, the alleviation of *S. pullorum-*induced compromised performance of laying hens following PE addition was likely associated with the improved gut microbiota, mainly characterized by the increased relative abundances of *Bacteroidetes* (*Bacteroides*), *Desulfovibrionaceae* and *Prevotellaceae*. Due to the limitation of the relative quantification of bacteria in analyzing gut microbiota-host interactions, the absolute quantification-based gut microbiota analysis might deserve to be further conducted, in order to validate the potential contribution of the modulated gut microbiota to the improved performance of laying hens following PE addition.

## Conclusion

Supplemental PE alleviated *S. pullorum*-induced impairment in production performance and intestinal disruption by attenuating intestinal inflammation and barrier dysfunction in laying hens, which could be partially responsible by the capability to remodel gut microbial composition, particularly the enrichment of SCFAs-producing bacteria. This study can expand our fundamental knowledge concerning the roles of gut microbiota in mediating the various physiological functions of PE in animals.

## Data Availability

The datasets generated for this study can be found in the Sequence Read Archive of the NCBI.

## Ethics Statement

The animal study was reviewed and approved by the Animal Care and Use Committee of the Feed Research Institute of the Chinese Academy of Agricultural Sciences.

## Author Contributions

W-wW implemented the experimental design and wrote the manuscript. H-jJ conducted the animal trial and performed the sample analyses. H-jZ and JW assisted with data analysis. S-gW and G-hQ contributed to the experimental design and preparation of the manuscript. All authors discussed the results and reviewed the manuscript.

## Conflict of Interest Statement

The authors declare that the research was conducted in the absence of any commercial or financial relationships that could be construed as a potential conflict of interest.
