# Supplementary material for: Supplemental Plant Extracts From Flos lonicerae in Combination With Baikal skullcap Attenuate Intestinal Disruption and Modulate Gut Microbiota in Laying Hens Challenged by Salmonella pullorum
Source: Front Microbiol. 2019 Jul 24;10:1681. doi: 10.3389/fmicb.2019.01681 (PMC6668501; doi:10.3389/fmicb.2019.01681)
Supplement: Supplementary file 1 [file Table_1.DOCX]

**Table S1**. Effects of plant extracts (PE) from *Flos Lonicerae* in combination with *Baikal Skullcap* on the α-diversity of ileal microbiota in laying hens at 3 d post *S. pullorum* infection (*n*=6)

|  | NC^†^ | PC | T | *P*-value |
| --- | --- | --- | --- | --- |
| Shannon index | 2.64±0.51 | 1.93±0.21 | 2.28±0.44 | 0.483 |
| Simpson index | 0.23±0.07 | 0.30±0.06 | 0.28±0.07 | 0.755 |
| Chao1 estimator | 331.11±17.14^a^ | 182.14±5.35^b^ | 206.37±30.97^b^ | <0.001 |
| ACE estimator | 326.74±16.51^a^ | 212.01±19.08^b^ | 225.07±27.81^b^ | 0.004 |

^a,b^ Values with different superscripts within the same row differ signiﬁcantly (*P* < 0.05).

^*^ NC, negative control (birds were free of challenge); PC, positive control (birds were challenged with *S. pullorum* at the end of wk 4 of the experiment); T, treatment group (PC + plant extracts treatment at 1000 mg/kg).
