# Supplementary material for: Supplemental Plant Extracts From Flos lonicerae in Combination With Baikal skullcap Attenuate Intestinal Disruption and Modulate Gut Microbiota in Laying Hens Challenged by Salmonella pullorum
Source: Front Microbiol. 2019 Jul 24;10:1681. doi: 10.3389/fmicb.2019.01681 (PMC6668501; doi:10.3389/fmicb.2019.01681)
Supplement: Supplementary file 2 [file Table_2.DOC]

**Table S2-A. Relative abundance of ileal microbes (top ten) at phylum level**

|  | NC | Sd | PC | Sd | T | Sd |
| --- | --- | --- | --- | --- | --- | --- |
| *Firmicutes* | 0.625 | 0.130 | 0.811 | 0.055 | 0.663 | 0.095 |
| *Proteobacteria* | 0.255 | 0.116 | 0.099 | 0.052 | 0.092 | 0.043 |
| *Epsilonbacteraeota* | 0.010 | 0.004 | 0.046 | 0.030 | 0.052 | 0.031 |
| *Actinobacteria* | 0.042 | 0.019 | 0.040 | 0.011 | 0.040 | 0.026 |
| *Bacteroidetes* | 0.024 | 0.011 | 0.002 | 0.001 | 0.116 | 0.099 |
| *Fusobacteria* | 0.001 | 0.000 | 0.000 | 0.000 | 0.020 | 0.020 |
| *Tenericutes* | 0.001 | 0.001 | 0.000 | 0.000 | 0.007 | 0.007 |
| *Chloroflexi* | 0.011 | 0.008 | 0.000 | 0.000 | 0.000 | 0.000 |
| *Acidobacteria* | 0.011 | 0.008 | 0.000 | 0.000 | 0.000 | 0.000 |
| *Cyanobacteria* | 0.003 | 0.001 | 0.001 | 0.000 | 0.001 | 0.000 |

Sd, standard deviation.

**Table S2-B. Relative abundance of ileal microbes (top ten) at class level**

|  | NC | Sd | PC | Sd | T | Sd |
| --- | --- | --- | --- | --- | --- | --- |
| *Bacilli* | 0.515 | 0.125 | 0.591 | 0.098 | 0.495 | 0.119 |
| *Clostridia* | 0.105 | 0.025 | 0.212 | 0.112 | 0.150 | 0.084 |
| *Gammaproteobacteria* | 0.214 | 0.114 | 0.099 | 0.052 | 0.085 | 0.045 |
| *Campylobacteria* | 0.010 | 0.004 | 0.046 | 0.030 | 0.052 | 0.031 |
| *Actinobacteria* | 0.035 | 0.015 | 0.040 | 0.011 | 0.037 | 0.026 |
| *Bacteroidia* | 0.024 | 0.011 | 0.002 | 0.001 | 0.116 | 0.099 |
| *Fusobacteriia* | 0.001 | 0.000 | 0.000 | 0.000 | 0.020 | 0.020 |
| *Alphaproteobacteria* | 0.033 | 0.023 | 0.000 | 0.000 | 0.001 | 0.000 |
| *Erysipelotrichia* | 0.001 | 0.001 | 0.005 | 0.003 | 0.007 | 0.007 |
| *Negativicutes* | 0.004 | 0.001 | 0.003 | 0.001 | 0.011 | 0.008 |

Sd, standard deviation.

**Table S2-C**. Relative abundance of ileal microbes (top ten) at family level

|  | NC | Sd | PC | Sd | T | Sd |
| --- | --- | --- | --- | --- | --- | --- |
| *Lactobacillaceae* | 0.460 | 0.130 | 0.545 | 0.104 | 0.446 | 0.114 |
| *Peptostreptococcaceae* | 0.060 | 0.029 | 0.194 | 0.115 | 0.094 | 0.081 |
| *Pasteurellaceae* | 0.075 | 0.041 | 0.096 | 0.052 | 0.074 | 0.047 |
| *Enterobacteriaceae* | 0.107 | 0.104 | 0.002 | 0.002 | 0.007 | 0.007 |
| *Enterococcaceae* | 0.047 | 0.038 | 0.021 | 0.007 | 0.011 | 0.004 |
| *Helicobacteraceae* | 0.006 | 0.004 | 0.013 | 0.006 | 0.040 | 0.029 |
| *Clostridiaceae_1* | 0.037 | 0.023 | 0.017 | 0.013 | 0.008 | 0.005 |
| *Campylobacteraceae* | 0.004 | 0.002 | 0.034 | 0.032 | 0.012 | 0.010 |
| *Bacteroidaceae* | 0.012 | 0.005 | 0.001 | 0.000 | 0.054 | 0.043 |
| *Micrococcaceae* | 0.008 | 0.006 | 0.020 | 0.008 | 0.007 | 0.004 |

Sd, standard deviation.

**Table S2-D**. Relative abundance of ileal microbes (top ten) at genus level

|  | NC | Sd | PC | Sd | T | Sd |
| --- | --- | --- | --- | --- | --- | --- |
| *Lactobacillus* | 0.460 | 0.130 | 0.545 | 0.104 | 0.446 | 0.114 |
| *Romboutsia* | 0.060 | 0.029 | 0.194 | 0.115 | 0.094 | 0.081 |
| *Gallibacterium* | 0.075 | 0.041 | 0.096 | 0.052 | 0.074 | 0.047 |
| *Escherichia-Shigella* | 0.106 | 0.104 | 0.002 | 0.002 | 0.007 | 0.006 |
| *Enterococcus* | 0.047 | 0.038 | 0.021 | 0.007 | 0.011 | 0.004 |
| *Helicobacter* | 0.006 | 0.004 | 0.013 | 0.006 | 0.040 | 0.029 |
| *Campylobacter* | 0.004 | 0.002 | 0.034 | 0.032 | 0.012 | 0.010 |
| *Candidatus_Arthromitus* | 0.037 | 0.023 | 0.017 | 0.013 | 0.005 | 0.004 |
| *Bacteroides* | 0.012 | 0.005 | 0.001 | 0.000 | 0.054 | 0.043 |
| *Staphylococcus* | 0.002 | 0.001 | 0.008 | 0.004 | 0.023 | 0.020 |

Sd, standard deviation.
