# Supplementary material for: Supplemental Plant Extracts From Flos lonicerae in Combination With Baikal skullcap Attenuate Intestinal Disruption and Modulate Gut Microbiota in Laying Hens Challenged by Salmonella pullorum
Source: Front Microbiol. 2019 Jul 24;10:1681. doi: 10.3389/fmicb.2019.01681 (PMC6668501; doi:10.3389/fmicb.2019.01681)
Supplement: Supplementary file 3 [file Data_Sheet_1.PDF]

A. Functional prediction based on KEGG orthologs

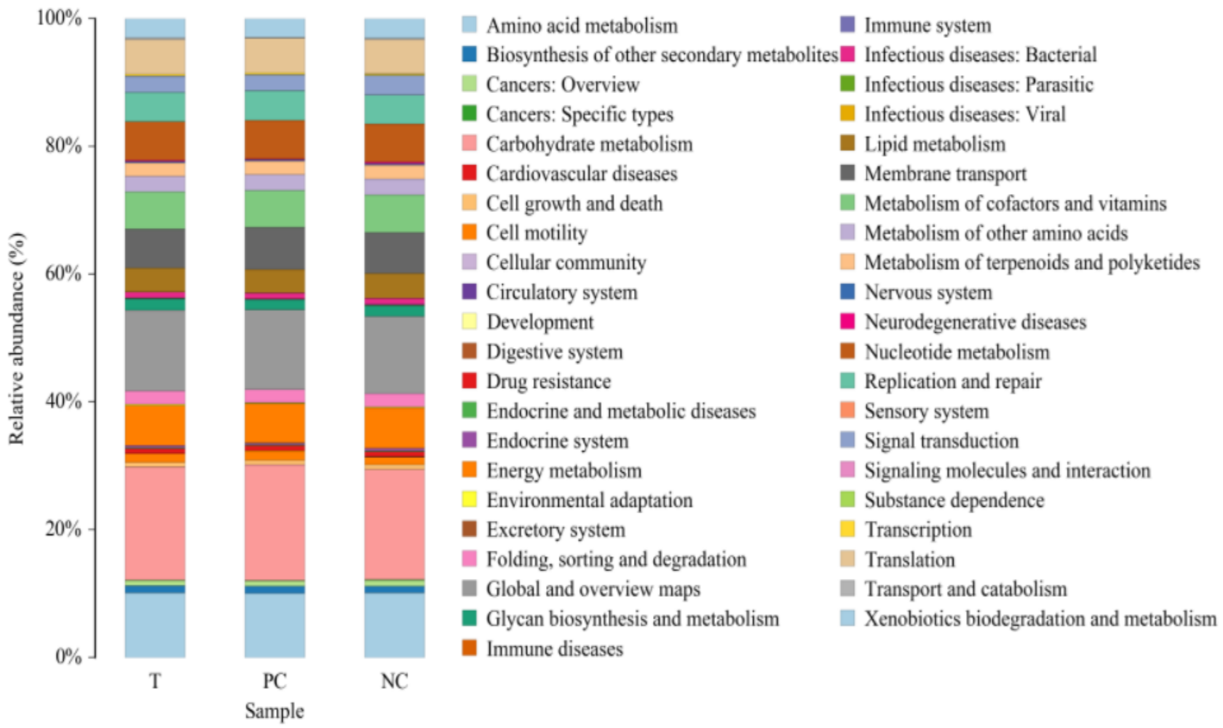

B. Functional prediction based on COG categories

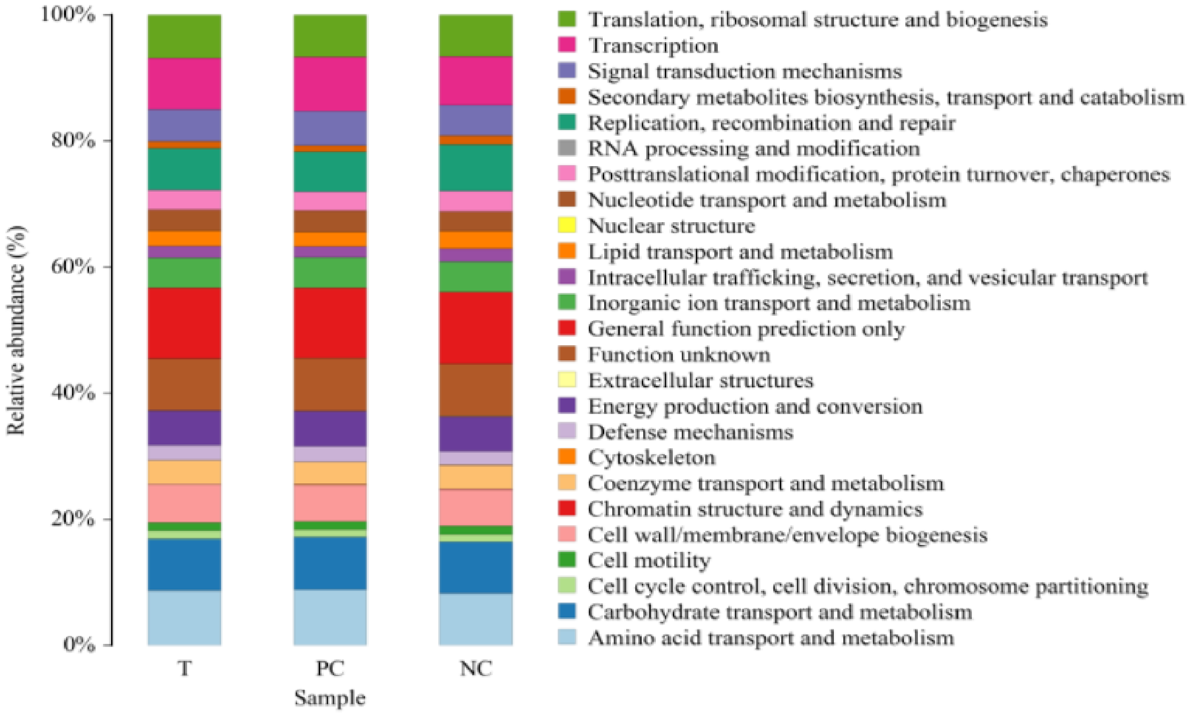

Figure S1. Functional prediction of ileal microbiota based on Kyoto Encyclopedia of Genes and Genome (KEGG) orthologs and Clusters of Orthologous Groups (COG) categories in laying hens at 3 d post S. pullorum infection (*n*=6). NC, negative control (birds were free of challenge); PC, positive control (birds were challenged with S. pullorum at the end of wk 4 of the experiment); T, treatment group (PC + plant extracts treatment at 1000 mg/kg).
